# Affecting patients with work-related problems by educational training of their GPs: a cost-effectiveness study

**DOI:** 10.1186/s12875-019-0924-9

**Published:** 2019-03-02

**Authors:** Cornelis de Kock, Cindy Noben, Antoine Lagro-Janssen, Peter Lucassen, André Knottnerus, Angelique de Rijk, Frans Nijhuis, Romy Steenbeek, Silvia Evers

**Affiliations:** 1 Department of Primary and Community Care, Gender and Women’s Health, Radboud University Nijmegen Medical Center, PO Box 9101, 6500 HB Nijmegen, The Netherlands; 2 Department of Health Services Research, CAPHRI School of Public Health and Primary Care, Maastricht University, Maastricht, The Netherlands; 3 Department of Primary and Community Care, Radboud University Nijmegen Medical Center, Nijmegen, The Netherlands; 4 Department of General Practice, Maastricht University, Maastricht, The Netherlands; 5 Department of Social Medicine, CAPHRI School of Public Health and Primary Care, Maastricht University, Maastricht, The Netherlands; 6 Department of Work and Social Psychology, Maastricht University, Maastricht, The Netherlands; 7 TNO Work, Health and Care, Leiden, The Netherlands; 8 Trimbos Institute, Netherlands Institute of Mental Health and Addiction, Utrecht, The Netherlands; 9 OOR ZON, Maastricht University Medical Centre, Maastricht, The Netherlands

**Keywords:** Health economics, Occupational/environmental medicine, Primary care, Randomized controlled trial, Rehabilitation/disabilities

## Abstract

**Background:**

Assessing the cost effectiveness of training aimed at increasing general practitioners’ (GP) work awareness and patients’ work-related self-efficacy and quality of life.

**Methods:**

A cluster randomized controlled trial in twenty-six GP practices in the southeast of the Netherlands with 32 participating GPs. GPs working in an intervention group practice received training and GPs working in a control group practice delivered usual care. The training intervention consisted of lectures and workshops aimed at increasing GPs’ work awareness and more proactive counseling for patients with work-related problems (WRP). Subjects were working age patients with paid work for at least 12 h per week, who visited one of the participating GPs during the study period. As outcome measures we used the Return to Work Self Efficacy scale to assess patients’ work-related self-efficacy and the Euroquol to assess quality of life. We also measured health care costs and productivity costs. With a 4-item questionnaire we asked patients to assess their GPs’ work awareness. Data were collected at baseline, after 6 and 12 months.

**Results:**

Data of 280 patients could be analyzed. The patient related outcomes did not improve after GP training. The change in GP work awareness and the overall mean cost difference (of €770) in favor of the intervention group were not significant.

**Conclusions:**

The training intervention presented in this paper was not cost-effective. Training which is further personalized and targeted at high risk groups with respect to WRP, is more likely to be cost effective.

**Electronic supplementary material:**

The online version of this article (10.1186/s12875-019-0924-9) contains supplementary material, which is available to authorized users.

## Background

Although most health professionals acknowledge the importance of their patient’s work, occupation and the ability to work often are not discussed during consultations. Even general practitioners (GPs), who are trained to deliver patient-centred care, accounting for the background and context of their patients, do not always pay attention to work-related health issues [1–3]. Nonetheless, a more active role of primary care providers with respect to their patients’ working context is valuable because of the beneficial relation between work and health. Previous studies have demonstrated this relation in both care- and cure settings [4–6]. In order to reduce practice variation, The Health Council of the Netherlands published a set of guidelines about the management of diseases which frequently lead to long term sickness absence [7]. And in the UK the Department of Work and Pensions (DWP) in 2010, replaced the sickness certificate by the “Fit Note” and more recently investigated the effect of telephonic support [8, 9]. When the Fit Note was introduced, the National Education Programme offered workshops developed by the Royal College of GPs which were attended by 2865 GPs. This programme resulted in an increased confidence managing consultations regarding work and health among the GPs [10]. When using the Fit Note, GPs can specify the way in which a patient’s work should be modified to make return to work possible [9]. Other strategies aimed to improve the collaboration between professionals working in curative health settings and those working in occupational health settings [11–13]. So far these strategies have had at best limited success [14, 15]. Moreover, if collaboration was achieved, it did not necessarily improve the outcomes with respect to return to work [16]. Studies using multifactorial analyses to find out which GP factors influence sick leave showed contradictory results and on the whole did not indicate GPs are an important factor [17–20].

For most GPs sickness certification or giving advice about work is not amongst their most preferred activities [21, 22]. This has been explained by the complexity of the task and by the fact that GPs tend to prioritise the interest of and relation with their patients, over those of other stakeholders [23, 24]. For Dutch GPs, an extra reason may be the fact that they, unlike their colleagues in other countries, do not have a formal role in sickness certification or in work rehabilitation [5, 25]. Still, GPs who want to deliver patient-centred care should structurally and proactively discuss paid work as one of the important context factors [26–28]. This is considered to be beneficial for several reasons: it can lead to better analyses of health problems and better tailoring of treatment strategies; GPs possibly recognise work-related problems (WRP) earlier and thus may prevent (long term) sick leave; the collaboration between primary care and other professionals involved in workers’ health may benefit from sharing a common agenda [2, 11]. In other studies it was demonstrated that training GPs can increase their patient centredness and help them to recognise and address difficult subjects like family violence [29, 30].

Therefore, we developed an educational GP training program aimed at making GPs more attentive to their patients’ work context and improve their management of WRP [31]. We performed this study to determine whether the GP training programme is cost-effective with respect to patient’s work-related self-efficacy and quality of life. We will also assess the effect on GPs’ work awareness. To assess the influence of potential confounding factors, gender aspects and work-related aspects were studied as well.

## Methods

An educational GP training program was developed to make GPs more attentive to the context of their patients’ work. We conducted a cluster randomized controlled trial (RCT) in which GPs were randomized to the intervention group or the control group. GPs working in the same practice were allocated to the same group to prevent contamination. Intervention group GPs received educational training, control group GPs provided usual care. The results with respect to the patient level and GP level outcomes were recently published [32]. This paper reports on the cost-effectiveness of our intervention with respect to work-related self-efficacy and quality of life. Detailed information regarding the methodology of this trial has been published elsewhere [31, 32].

### Intervention and usual care

GPs in the intervention group received accredited 5-h training which was based on the findings of previously conducted focus groups [33]. Seven items were covered in lectures and interactive workshops during the training: (i) the societal relevance of a proactive approach of the connection between work and health by GPs; (ii) reflection by the participants on their ‘usual care’ for WRP and barriers to provide care for WRP; (iii) legislation regarding work and absenteeism, the role of occupational physicians (OPs) and collaboration between GPs and OPs; (iv) gender aspects of work and WRP; (v) demonstration of good practice for the management of WRP, consisting of an activating approach, scheduling at least one follow-up visit and counselling, for instance about a timely discussion of problems with a supervisor or OP rather than staying home without a plan; (vi) structural registration of work-related data in the electronic medical files including adequate coding according to the International Classification of Primary Care (ICPC); and (vii) information about study logistics and data collection. Three months after the initial training a three hour booster training took place [31]. GPs included in the control group provided care as usual. After the study, GPs in the care as usual group were also offered the educational training as an incentive for adhering to the study protocol [31].

### Patient population

Between February 2012 and January 2013 all patients visiting participating GPs were invited by the receptionist to complete a short questionnaire to check the inclusion criteria. Patients were included if they: (i) were 18–63 years of age, (ii) had paid work for at least twelve hours per week, (iii) sufficiently understood the Dutch language and (iv) had given their informed consent. Patients were excluded from the study if more than twelve months had elapsed between completions of two consecutive questionnaires. Furthermore, at least two out of three measures on the Return To Work Self-Efficacy scale (RTW-SE) had to be completed in order to provide a meaningful interpretation for the economic analyses on the impact of the training on patients. After informed consent participants received the first questionnaire (see Additional file 1). After at least six months a second questionnaire followed and after at least another six months the final questionnaire had to be completed. Depending on the patient’s preferences, paper or online questionnaires were handed out or mailed to them.

### Outcome measures

The main outcome for the cost-effectiveness analysis was work-related self-efficacy measured by the eleven item Return-to-Work Self-Efficacy (RTW-SE) scale. This scale has been validated in sick-listed patients in whom it predicted return to work. It measures the extent to which people feel able to handle the demands of their job using 11 items, which can be rated from ‘1’ (completely disagree) to ‘6’ (completely agree) [34]. The total score ranges from 1 to 6, with higher values indicating better work-related self-efficacy. As we included patients with paid work, rather than patients on sick leave, we called the construct we measured “work-related self-efficacy”, rather than “return to work self-efficacy”.

The main outcome for the cost-utility analysis was quality adjusted life years (QALY), measured by using the EQ-5D-5 L. This questionnaire measures the patient’s health state of 5 dimensions on 5 levels ranging from no problems to extreme problems. The dimensions are mobility, self-care, usual activities, pain/discomfort, and anxiety/depression. The score ranges from 5 to 25, with higher scores indicating worse functioning. To estimate the utility of health states described by the patients, the EQ-5D-5 L crosswalk value set with the Dutch tariff was used [35]. Higher QALYs indicate more improvement in quality of life.

GP work awareness was measured with the self-developed GP work awareness scale (GWAS). In this 4 item scale, patients are asked whether the GP knows their occupation, discusses a possible relation between their health problem and their work, discusses sick leave, and helps to find solutions for any WRP. Each item can be rated ‘1’ (agree) or ‘0’ (not agree) and thus the scale ranges from 0 to 4.

### Cost measures

Cost data were collected from a societal perspective meaning that all costs and benefits will be captured independently of those who bear the costs or those who receive the benefits [36]. All costs were measured from 2012 onwards (start of handing out the first questionnaires) and therefore adjusted to the index year 2012 using consumer price indices. In the questionnaires we asked how often, over the last 6 months, the participants had taken sick leave, visited different health care professionals, were admitted to hospitals, received informal care, and multiplied these frequencies with the standard costs of sick leave and prices of care. By adding these components we calculated the costs. All costs where categorized and calculated in 4 main cost categories: intervention, healthcare, patient and family, and productivity costs. More details on the measurement and valuation of the costs incorporated in the cost categories can be found in the Supplementary Data Table S1 (see Additional file 2).

### Statistical and economic analyses

Baseline characteristics were compared for both groups by using analysis of variances (Anova), non-parametric Kruskal-Wallis analysis for continuous variables or Pearson’s chi square test for categorical variables. We calculated the intra cluster correlation coefficients (ICC) of the RTW-SE, QUALY, health care costs and productivity costs. For the GWAS, we used an ANCOVA model with follow-up score as dependent variable and baseline score and study condition as independent variables to assess the effect of the intervention.

The differences in costs were calculated between both groups at the different measurement periods. Because cost distributions are often highly skewed, bootstrapping with 1000 replications was used to estimate bootstrap confidence intervals around the cost differences. Missing data were handled using SPSS missing value analysis on item level (mean imputation).

The differences in costs (incremental costs) were divided by the incremental effects, resulting in incremental cost effectiveness ratios (ICER). Non-parametric bootstrapping was also used to estimate the uncertainty surrounding the ICER (5000 replications). Afterwards, the bootstrapped cost effect pairs (i.e. ICERs) were plotted on the cost effectiveness planes.

Five sensitivity analyses were conducted to assess the robustness of the findings. First, 4 subgroup analyses were conducted to test for participant heterogeneity by assessing gender differences among patients and GPs. In the fifth sensitivity analysis changed working hours from baseline to 12 months follow-up served as the effect measure.

## Results

### Baseline characteristics

**Fig. 1:**
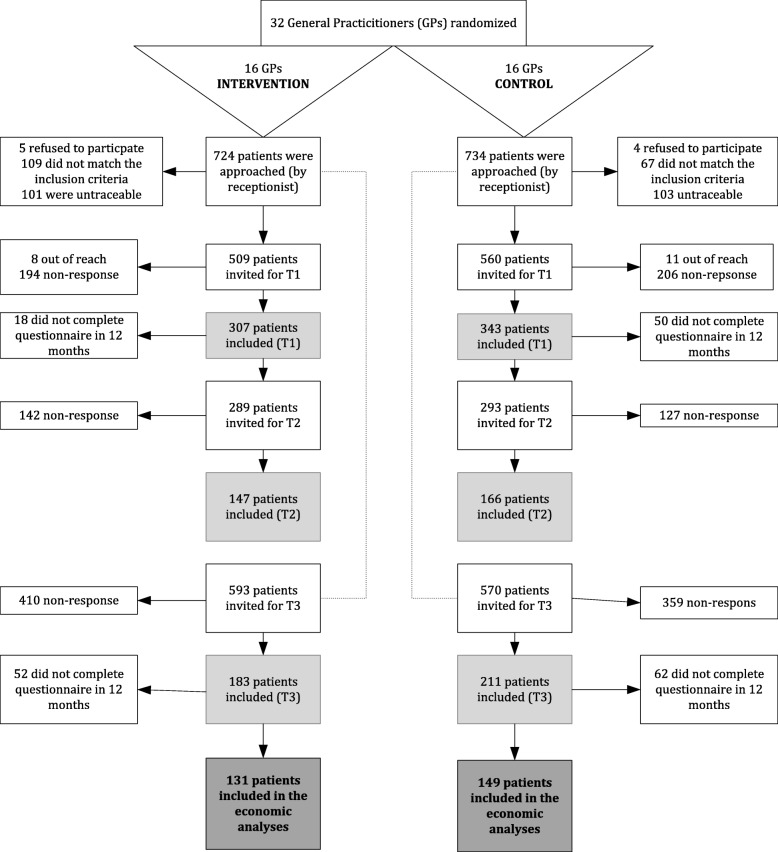
Flowchart showing the inclusion of patients for the economic evaluation (*n* = 280)

After 12 months follow-up, 280 patients were included in the economic evaluation: 131 in the intervention group and 149 in the control group (see Fig. 1).

The population characteristics are presented in Table 1. Most patients included in the study were female and permanently employed. The number of days absent from work in the past 6 months was higher in the intervention group but this difference was not statistically significant (*p* = 0.07). The average health care utilization costs were highest in the control group. All other cost categories were highest for the intervention group. At baseline, productivity costs accounted for the largest part of the total costs in both groups, with a significant difference in absenteeism costs between the groups. The ICC of the RTW-SE was 0.05, the ICCs of Utility, health care costs and productivity costs were all < 0.001.

**Table 1:** Baseline characteristics (*N* = 280) (year of financial data = 2012)

	Intervention (*n* = 131)	Control (*n* = 149)	*p*-value
Age, mean (sd)	45.14 (10.01)	46.41 (10.96)	0.31 (a)
Gender, *N* (%)			
Female	83 (63)	86 (58)	0.34 (b)
Male	48 (37)	63 (42)	
Education, *N* (%)†			
Low	5 (4)	6 (4)	0.67 (b)
Intermediate	85 (65)	98 (66)	
High	41 (31)	45 (30)	
Working hours, mean (sd)	29.41 (10.39)	31.04 (11.68)	0.22 (a)
Job contract, *N* (%)			
Entrepreneur	6 (5)	13 (9)	0.21(b)
Employee (permanent employment)	99 (76)	114 (77)	
Employee (temporary employment towards permanent employment)	8 (6)	7 (5)	
Employee (temporary employment/fixed term)	12 (9)	10 (7)	
Interim temporary worker	1	1	
On call worker/substitute	0	3 (2)	
Social Employment Law worker	5 (4)	1	
Supervisor, *N* (%)			
One	116 (89)	130 (87)	0.74 (b)
More than one	15 (11)	19 (13)	
Managerial position, *N* (%)	39 (30)	40 (27)	0.59 (b)
Shift work, *N* (%)	29 (22)	30 (20)	0.83 (b)
Job sector, *N* (%)			
Craft and industry	15 (11)	25 (17)	0.46(b)
Transport	8 (6)	9 (6)	
Administration	20 (15)	12 (8)	
Commercial	14 (11)	22 (15)	
Services	18 (14)	23 (15)	
Heath and care	32 (24)	33 (22)	
Teaching	11 (8)	7 (5)	
Specialist (discipline, expertise)	10 (7)	15 (10)	
Agriculture	3 (2)	3 (2)	
Self-rated general health^¥^, mean (sd)	72.69 (21.5)	75.6 (21.3)	0.26 (a)
Long term disability, N (%)	51 (40)	61 (39)	0.73 (b)
Days absent from work past 6 months, mean (sd)	15.14 (30.65)	8.34 (18.43)	0.07 (a)
EQ-5D-5 L Utilities, mean (sd)^₸^	0.83 (0.16)	0.84 (0.15)	0.73 (a)
RTW-Self efficacy, mean (sd)	4.74 (1.29)	4.77 (1.15)	0.83 (a)
Costs, mean in € (sd)			
Health care utilization	452.74 (647)	562.28 (2079)	0.56 (c)
Medication and aids	54.04 (135)	47.74 (114)	0.47 (c)
Patient and family	46.83 (96.11)	38.47 (77.41)	0.42 (c)
Informal care	11.03 (66.9)	5.97 (34.47)	0.42 (c)
Travel and parking	35.8 (46.07)	32.5 (61.88)	0.62 (c)
Productivity	3906.58 (10,878.11)	2201.14 (5208.97)	0.09 (c)
Absenteeism	2426.83 (5555.2)	1245.96 (3403.87)	0.36 (c)
Presenteeism	1479.75 (6312.95)	955.18 (3205.42)	0.37 (c)

†Low = pre- or primary school; intermediate = lower- or upper secondary; high = tertiary school, university, or postgraduate ¥ On a rating scale from 0 (worse health) to 100 (perfect health) ₸High scores indicate better perceived health outcomes (a) Anova (b) Pearson Chi-squared (c) Non-parametric Kruskal Wallis

*Significant at the 5% level

### Outcome analysis

The mean RTW-SE score at baseline was comparable and did not differ significantly (*p* > 0.05) between the intervention and the control group. Over time, scores on RTW-SE were comparable (at six-month follow-up control group 4.95 and intervention group 4.86; twelve months follow-up control group 4.9 and intervention group 4.92).

Quality of life (utilities) at baseline was similar and did not differ significantly (*p* > 0.05) between the intervention and the control group. The change in quality of life after six-month follow-up (control group 0.86 and intervention group 0.86), and twelve months later (control group 0.86 and intervention group 0.87) was small. Quality of life was comparable for the intervention group (0.87; standard deviation (SD) 0.1) and the control group (0.86; SD 0.13), and did not differ significantly (*p* = 0.53).

The estimated marginal means of the GWAS for the intervention group GPs was 2.5 (95% confidence interval (CI): 2.19–2.79) and for the control group GPs 2.1 (95% CI: 1.82–2.44) (*p* = 0.1).

### Cost analysis

The intervention costs were almost negligible (€0.48). Healthcare costs for the 12-months follow-up were higher in the control group, both for health care utilization and medication usage. The mean cost difference of €945 was in favor of the intervention group. Also within the control group, more patient and family costs were made with a mean cost difference of €82 in favor of the intervention group. The productivity costs continued to account for the largest part of the mean total costs in both the control and intervention group. The difference in productivity costs between both groups (€330) was in favor of the control group. In total, the bootstrapped total mean costs yielded an incremental difference of €697 in favor of the intervention group (Table 2).

**Table 2:** Total costs per cost category after 12 months follow-up (*N* = 280) (year of financial data = 2012)

Cost category	Intervention group(*), costs €	Control group(#), costs €	Incremental costs, €	2.5–97.5 percentile
Intervention	0.48	0		
Health care utilization	1130.98	2082.99		
Medication and aids	52.14	72.82		
Bootstrapped subtotal, mean	1177.74	2122.38	−945	− 2721–184
Patient and family				
Informal care	34.04	94.2		
Parking and travel	52.62	73.41		
Bootstrapped subtotal, mean	86.39	168.37	−82	−207 - 9
Other sectors (productivity)				
Absenteeism	2675.22	2344.45		
Presenteeism	2214.72	2263.03		
Bootstrapped subtotal, mean	4936.95	4607.14	330	− 2475 - 3113
Bootstrapped total, mean	6168.72	6865.56	−697	−4351 - 2936

Note: Bootstrapped subtotal means might deviate due to rounding

(*) Based on n = 131 (#) Based on n = 149

### Incremental cost effectiveness ratio

**Fig. 2:**
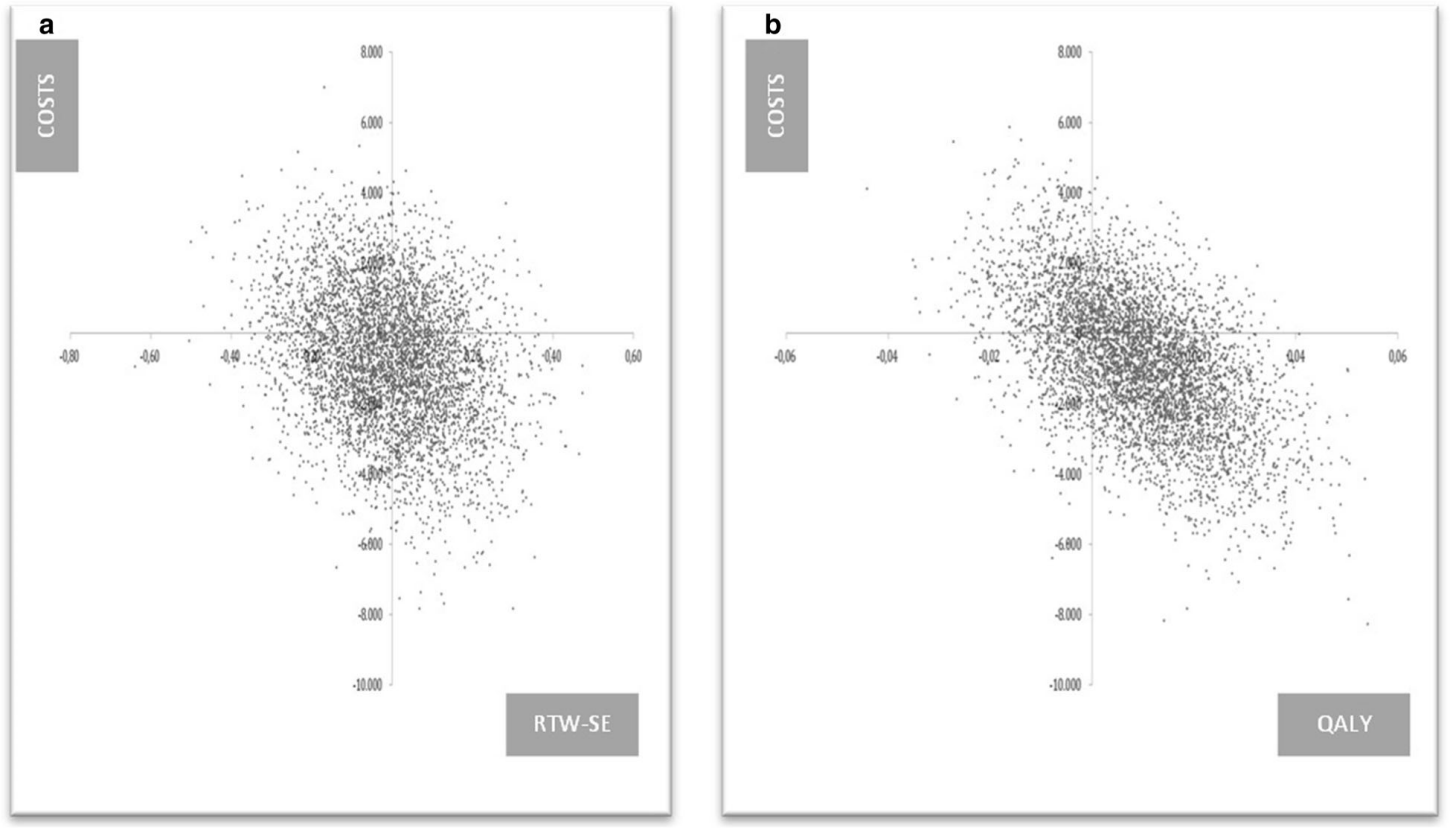
Cost effectiveness planes illustrating (**a**) the cost effectiveness for work-related self efficacy (RTW-SE) among patients and (**b**) the costs utility for quality of life (QALY) (*N* = 280; financial data = 2012) **a** Cost effectiveness plane representing the uncertainty around the mean incremental costs and mean incremental effects (RTW-SE) of the intervention compared with the control condition. **b** Cost effectiveness plane representing the uncertainty around the mean incremental costs and mean incremental effects (QALY) of the intervention compared with the control condition

Based on the bootstrapped ICERs, no meaningful ICER for the cost effectiveness analysis was found. The ICERs are distributed almost equally among all the four quadrants of the plane (Fig. 2_a). The bootstrapped ICERs on the plane for the cost utility analysis (Fig. 2_b**)**, presents similar results and demonstrates the harshness to correctly interpret the negative ICER (− €87,214.28) because of the almost equal distribution of the cost effect pairs along the four quadrants.

No differences in RTW-SE between the intervention and control group were found (Table 3). A mean cost difference of € 770 (95% CI: € -2936 - € 4351) was detected in favor of the intervention group.

**Table 3:** Mean cost and effect differences between the intervention and control group including incremental cost effectiveness and cost utility ratios, and cost effectiveness plane distributions

	Effect measure	Sample Size		∆Cost (€2012)	∆Effect	ICER	Distribution (%) CEA plane (quadrant)			
		INT	CTR				NE	SE (dominant)	SW	NW (inferior)
Main analysis										
Cost Effectiveness	RTW-SE	131	149	−770.67	0	168,376.09	13	35	29	13
Cost Utility	QALY	131	149	−770.67	0.01	−87,214.28	19	56	9	16
Subgroup & Sensitivity analysis										
Female GPs	RTW-SE	46	57	− 2751.1	0.05	−50,730.37	7	52	33	8
Male GPs	RTW-SE	84	92	338.9	−0.06	− 5745.96	16	21	23	40
Female patients	RTW-SE	83	86	− 2914.6	−0.03	111,755.92	2	41	50	7
Male patients	RTW-SE	48	63	2693.3	0.01	415,744.72	38	14	6	42
Hours work	Working hours	131	149	−770.67	0.04	−17,655.77	25	52	14	9

INT = intervention group | CTR = control group

NE = north-east quadrant of the cost effectiveness plane, which indicates the intervention is more effective and more costly than the control condition | SE = south-east quadrant of the cost effectiveness plane, which indicates the intervention is more effective and less costly than the control condition | SW = south-west quadrant of the cost effectiveness plane, which indicates the intervention is less effective and less costly than the control condition | NW = north-west quadrant of the cost effectiveness plane, which indicates the intervention is less effective and more costly than the control condition

Results of the sensitivity analyses are also shown in Table 3. The 4 gender subgroups show no probability of the intervention becoming cost effective when studying RTW-SE among female or male patients, nor on the influence of female or male GPs on RTW-SE among their patients. However, based on the findings in the subgroup analyses, it could be suggested that if a positive effect of the intervention on RTW-SE could be found, female patients might be more sensitive to this effect in comparison to male patients. It might also be suggested that this is the case for female GPs; if patient’s RTW-SE is attributable to the effort of GPs, female GPs might be more sensitive to achieve a positive effect on RTW-SE. The final sensitivity analysis, using work hours as an outcome measure, did not reveal other results.

## Discussion

In this study we were able to analyse data of 280 patients recruited from the waiting room population of 16 intervention group GPs and 16 control group GPs. We found no effect of our intervention on the patients’ work-related self efficacy or on their quality of life and no significant difference on societal costs or on the GPs’ work awareness.

### Comparison with other studies

The subject of work-related problems in general practice has been widely studied [15–20]. In most countries GPs have an important role in sickness certification. Our study was done among Dutch GPs who do not certify sickness. It corroborates the findings of the studies which used multifactorial analyses and concluded that GPs so far appear to have little influence on societal costs resulting from sickness absence.

Concerning the effect of training on GP behaviour we found several studies which demonstrated that training effectively changed GP behaviour, resulting in more patient-centered care and in patients being more satisfied [29, 30, 37]. Why have these studies been effective while ours was not effective? We consider extensive tailoring and continuous feedback as important elements for behaviour change in GPs [38]. These elements have been absent in our study. We expected our intervention to be beneficial for patients because of studies done in patients with mental health problems. Paying extra attention to work during their treatment, facilitated the return to work in patients with sickness absence because of common mental disorders and severe depression [39, 40]. It was also demonstrated that work-related self efficacy predicts return to work [34]. Therefore, we hypothesized that having GPs pay more attention to their patients’ work, might result in an increase in their patients’ work-related self efficacy, also in patients with work-related problems who had continued working. That we did not find this in our study could be explained by the ceiling effect as, compared to other patients in which this instrument was used, the average RTW-SE score of the participating patients in our study was relatively high [34].

### Implications for future research

For most workers, the GP practice is the point of access to the health care system but WRP can linger on for a long period of time without being recognised or addressed by GPs. Therefore, more effort should be put in strengthening the role of GPs with respect to the work of their patients. Future primary care-based studies should target subgroups of patients with an increased risk of WRP, e.g. patients with a chronic illness or mental health problem. In order to be successful, it is further recommended to use tailored implementation strategies providing both individualised and team level feedback [41]. In order to provide such feedback, outcome measures need to be selected and if needed developed, to align with expected effects of the intervention itself.

### Strengths and limitations

This is the first RCT studying the cost effectiveness of training to increase the recognition and improve the management of WRP by GPs. Outcome measures were patients’ work-related self-efficacy, quality of life and health care and productivity costs and GPs’ work awareness. Other strengths of this study are the fact that it was conducted in routine daily clinical practice with data collected from workers recruited from the GP waiting room population, limiting potential selection bias of patients with absenteeism or WRP. Additional strengths are the successful randomization resulting in comparability of the intervention and control groups with respect to most characteristics, the inclusion of a broad measure for productivity costs [36] and the assessment of gender differences [42, 43]. Furthermore, the economic evaluation was carried out from a societal perspective.

Several limitations of our study need to be considered that may inform future studies: to start, we did not succeed in including a sufficient number of patients, meaning that we could not limit follow up to patients with WRP or chronic illness. This may have resulted in the ceiling effect, which made it difficult to detect an effect. A lack of tailoring of our intervention is another limitation. Offering individualised feedback and assistance in identifying and overcoming barriers might have helped the trained GPs to improve their performance. Another limitation that needs consideration is that the cost and effect data were obtained via patient self-reported retrospective questionnaires which may have caused recall bias, potentially over- or underestimating true healthcare utilization and healthcare costs. As there is no ‘gold standard’ for measuring healthcare utilization, the method used in this study provides at least crude estimates of actual usage [44]. Finally, the time span between the measurements occasionally reached over 12 months due to practical and logistical reasons, but the impact of potential recall bias was assumed to be equal for both groups due to the robust randomisation procedure.

## Conclusion

Our training aimed at increasing GP work awareness and improving counselling for patients with WRP, did not result in an increased work-related self efficacy or quality of life among patients, nor did it significantly reduce societal costs or increase the work awareness among the GPs. It is expected that targeting at specific patient groups and tailoring of the intervention to individual GPs’ needs will improve the intervention. Further, expected effects that align with the intervention content specifically should be tested.

## Additional files

Additional file 1: “Questionnaire GPs@Work” Description: English language version of the questionnaire we have used in our study. (DOCX 115 kb)

Additional file 2: **Supplementary Data Table S1. **Description: Cost calculations per cost category (year of financial data = 2012). (DOCX 24 kb)

Additional file 3: “Ethics waiver” Description: Letter from the institutional ethics review board concluding that approval was not needed according to Dutch law. (DOCX 11 kb)

## Abbreviations

ANCOVA: Analysis of covariance
EQ-5D-5 L: EuroQoL 5 Dimensions 5 Levels
GP: General practitioner
GWAS: GP work awareness scale
ICC: Intra cluster correlation coefficients
ICER: Incremental cost effectiveness ratio
ICPC: International Classification of Primary Care
OP: Occupational physicians
QALY: Quality adjusted life years
RCT: Randomized controlled trial
RTW-SE: Return To Work Self-Efficacy
WRP: Work-related problems
