# Supplementary material for: Affecting patients with work-related problems by educational training of their GPs: a cost-effectiveness study
Source: BMC Fam Pract. 2019 Mar 2;20:38. doi: 10.1186/s12875-019-0924-9 (PMC6397438; doi:10.1186/s12875-019-0924-9)
Supplement: Supplementary file 1 — “Questionnaire GPs@Work” Description: English language version of the questionnaire we have used in our study. (DOCX 115 kb) [file 12875_2019_924_MOESM1_ESM.docx]

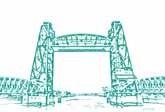

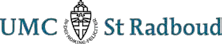

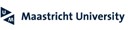

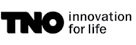


**H u i s a r t s e n**

***w e r k***

**Questionnaire**

**Work & Health**

Dear participant,

We greatly appriciate your willingness to contribute to our research by completing this questionnaire.

We investigate how general practitioners can take your working situation into account when you consult them. Work is important because it can be beneficial or detrimental for your health. If your GP pays attention to your working situation your health may benefit. We investigate the way your GP pays attention to your work and how this possibly could be improved.

This questionnaire consists of questions about your personal details, about your work, your health and your health care utilisation.

You may wonder why certain questions are part of this questionnaire. However, every question is important for our study. Therefore it is important that you answer the questions as accurately as possible and answer all questions. Please choose the answer which best applies to your situation.

## Confidentiality

Your answers will be dealt with utmost confidentiality. Only members of our research team will have access to these data. The data will not be shared with your general practitioner or occupational physician. The data will only be used for our study. Your data will be coded so you will never find your name in a report of our study.

## Time necessary to complete questionnaire

This questionnaire consists of 55 items. Completing the questionnaire will take about 20 minutes.

## Contact

If you have any questions about this questionnaire or about our study, please do not hesitate to contact the researcher:

Kees de Kock, general practitioner in Deurne

E-mail: k.dekock@elg.umcn.nl

Telefoon: (024) 366 80 36

UMC St Radboud

Vakgroep Huisartsgeneeskunde Vrouwenstudies Medische Wetenschappen

**We wish to express our gratitude for your willingness to participate in our study!**

Kees de Kock, GP-researcher, UMC St Radboud

Prof. dr. Toine Lagro-Janssen, UMC St Radboud

Prof. dr. André Knottnerus, Universiteit Maastricht

## Your visit

Below are the first questions. These questions deal with the visit you have just paid to your general practitioner.

**1) Does your GP know your present employment or occupation?**


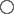
 Yes


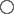
 No

**2) Do you think it is possible that the health problem for which you visited your GP is related to your work?**


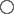
 Yes


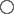
 No *(ga verder naar vraag 6)*

**3) Has the possibility of a relation between your health problem and your work been discussed?**


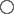
 Yes


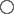
 No

**4) Did you discuss whether it is advisable to work with your health problem?**


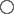
 Yes


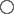
 No


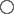
 Not applicable

**5) Did your GP help you to find solutions to deal with work-related problems?**


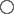
 Yes


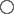
 No


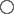
 Not applicable

## Your opinion

We would also like to know your opinion about what GPs in general should do with respect to their patients’ work. The following statements deal with this issue.

**6) Please, after the following statements, check the answer which best represents your opinion.**

Please choose the applicable option for each item:

|  | **Completely agree** | **Mostly agree** | **No opinion** | **Mostly disagree** | **Completely disagree** |
| --- | --- | --- | --- | --- | --- |
| I think it is important that GPs know the occupation of their patients. | 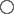 | 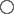 | 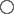 | 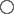 | 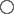 |
| I think it is important that the GP advises me about going to work or taking sick leave. | 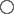 | 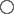 | 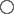 | 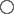 | 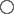 |

**7) Which approach towards patients’ work would you think most fitting for your GP? Please describe below.**

………………………………………………………………………………………………………………………………………………..………………………………………………………………………………………………………………………………………………..………………………………………………………………………………………………………………………………………………..………………………………………………………………………………………………………………………………………………..………………………………………………………………………………………………………………………………………………..………………………………………………………………………………………………………………………………………………..…………………………………………………………………………………………………………………………………………………………………………………………………………………………..

## Expectations about working

**8)** The following statements are about your expectations about working. Quite possibly you are presently working your normal hours. It is also possbile that you are taking sick leave or only working a part of your normal hours. That does not matter for these questions. Please imagine the situation that you are working your normale hours (**with your present level of health complaints**).

Choose the applicable answer for each component:

| **If I go to work tomorrow I expect that:** | **Completely disagree** | **Mostly**  **disagree** | **Somewhat disagree** | **Somewhat agree** | **Mostly agree** | **Completely agree** |
| --- | --- | --- | --- | --- | --- | --- |
| I will be able to handle setbacks well. | 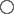 | 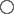 | 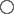 | 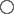 | 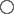 | 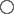 |
| Despite my emotions I will be able perform my tasks very well. | 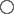 | 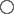 | 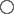 | 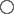 | 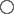 | 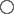 |
| I will be able to guard my limits. | 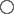 | 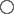 | 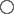 | 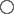 | 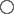 | 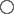 |
| I will be able to carry out my duties. | 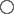 | 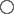 | 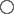 | 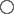 | 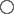 | 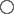 |
| I will be able to handle emotionally demanding situations. | 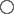 | 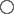 | 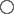 | 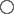 | 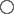 | 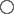 |
| I will have enough energy left for something else. | 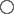 | 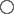 | 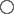 | 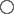 | 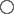 | 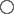 |
| I will be able to concentrate sufficiently on my work. | 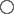 | 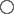 | 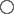 | 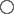 | 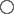 | 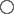 |
| I will be able to handle the strain at work. | 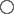 | 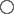 | 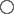 | 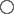 | 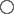 | 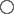 |
| I will be able to solve possible problems at work. | 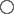 | 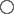 | 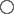 | 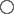 | 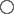 | 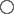 |
| I will be able to motivate myself enough to do my job. | 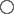 | 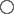 | 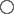 | 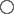 | 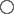 | 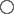 |
| I will be able to meet the physical requirements of my work. | 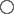 | 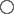 | 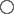 | 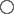 | 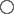 | 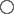 |

## Education

The next question deals with your education.

**9) What is the highest level of education which you completed?**


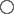
 No education completed


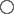
 Basic education


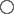
 Lower General Secondary Education


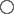
 Pre-vocational secondary education


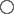
 Higher General Secondary Education, pre-university education, intermediate vocational education


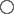
 Higher professional education


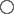
 University

## Occupation, work situation and income

The next questions are about your occupation, work situation and income.

**10) What is your working contract?**


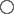
 Entrepreneur (with or without employees)

Worker with permanent contract

Worker with temporary contract which may be turned into a permanent contract.

Worker with temporary contract.

Temporary agency worker

On call work/substitute

Working in sheltered employment (e.g. in social workplace).

I am not gainfully employed (please continue to question 53)

**11) How many hours per week do you work on average?**
*If you have a contract, please indicate the number of hours you work according to your contract (not counting overtime).*

….. hours

**12) Within your paid work, do you have more than one Customer or employer?**

No, I have only one Customer/employment

Yes, I have more than one Customers/employments

**If you have more than one employments or Customers, please complete the questionnaire thinking of the employment in which (or Customer for whom) you work most hours.**

**13) Do you have the supervision of employees or colleagues?**

Yes

No

**14) Do you have irregular work, or do you work shifts?**

Yes

No

**15) Please indicate to which sector your occupation belongs.**

Crafts and industries (eg. painters, plumbers, mechanics, workers in food industry, builders, carpenters)

Transports (e.g. chauffeurs, loaders, train drivers)

Administration (e.g. accountancy)

Commerce (e.g. sales representatives, sales people, retail staff, realtors)

Services (e.g. cooks, waiters, cleaners, police, guards, hair dressers)

Healthcare and aid workers (e.g. doctors, nurses, social workers)

Education (teachers, all levels)

Professionals (e.g. architects, mathematicians, artists)

Agricultural and fisheries (farmer, cattle breeder, horticulturists, fishermen)

Other, namely…………………………………………………………………..

**16) What is your own average monthly income?**

*It is the salary you monthly receive. We are only asking for your income, not counting the income of any partner.*

< 1000 Euro’s per month

1000 - 2000 Euro’s per month

2000 - 3000 Euro’s per month

> 3000 Euro’s per month

I do not know

I do not want to tell

*Tip: you can divide your net year income by 12.*

## Health situation and work

The following questions are about your health situation and how it influences your work.

**17) How, in general, would you rate your health?**

Excellent

Very good

Good

Fair

Poor

**18) Do you have a chronic disease, illness or handicap?**

Yes

No *(please continue with question 21)*

**19) Which of the following chronic diseases, illnesses or handicaps trouble you most?**

Problems with arms or hands (also osteoarthritis, rheumatoid arthritis, RSI)

Problems with legs or feet (also osteoarthritis, rheumatoid arthritis, RSI)

Problems met back and neck (also osteoarthritis, rheumatoid arthritis, RSI)

Migraine or chronic/severe headache

Cardiovascular disease

Asthma, bronchitis, emphysema, Chronic obstructive pulmonary disease (COPD)

Gastrointestinal disorders

Diabetes

Severe skin diseases

Mental health problems/illness

Hearing problems

Epilepsy

A life threatening disease (eg. cancer, AIDS)

Visual handicap

Other, namely…………………………………………………

**20) Do you have any other chronic disease(s), illness(es) or handicap(s)?**

No

Yes, namely…………………………………………………

## Work functioning

The following questions are about the days during which you did (still) perform paid work. The questions deal with your functioning during your work, more specifically with the quantity and quality of the work you did.

**21) On how many working days during the last 6 months, could you get less work done and work less well, compared to a day during which you could perform well?**

During the last 6 months I could always work equally well

I could get less work done and/or work less well on the following number of days: ……..

*If you could work less or less well please write down the number of days during the last 6 months when this was the case.*

**22) How much work you have done during your last working day, during normal working hours, compared to a normal working day. Indicating “0” means you could do nothing whereas indicating “10” means you could do your normal amount of work.**

Choose the applicable answer for each item:

| Nothing 0 | 1 | 2 | 3 | 4 | 5 | 6 | 7 | 8 | 9 | Usual amount 10 |
| --- | --- | --- | --- | --- | --- | --- | --- | --- | --- | --- |
|  |  |  |  |  |  |  |  |  |  |  |

**23) On the scale below you can indicate the quality of the work you during your last working day compared to the normal quality. A “0” means your work was of very poor quality whereas a “10” indicates the same quality as normal.**

Choose the applicable answer for each item:

| Very bad quality 0 | 1 | 2 | 3 | 4 | 5 | 6 | 7 | 8 | 9 | Same quality as usual  10 |
| --- | --- | --- | --- | --- | --- | --- | --- | --- | --- | --- |
|  |  |  |  |  |  |  |  |  |  |  |

## Health status

The following questions are about your present health status.

**24) Which statement best describes your health TODAY?**

**(MOBILITY)**

I have no problems with walking about

I have slight problems with walking about

I have moderate problems with walking about

I have severe problems with walking about

I am unable to walk about

**25) Which statement best describes your health TODAY?**

**(SELF-CARE)**

I have no problems washing or dressing myself

I have slight problems washing or dressing myself

I have moderate problems washing or dressing myself

I have severe problems washing or dressing myself

I am unable to wash or dress myself

**26) Which statement best describes your health TODAY?
(USUAL ACTIVITIES** *e.g. work, study, housework, family or leisure activities)*

I have no problems doing my usual activities

I have slight problems doing my usual activities

I have moderate problems doing my usual activities

I have severe problems doing my usual activities

I am unable to do my usual activities

**27) Which statement best describes your health TODAY?
(PAIN / DISCOMFORT)**

I have no pain or discomfort

I have slight pain or discomfort

I have moderate pain or discomfort

I have severe pain or discomfort

I have extreme pain or discomfort

**28) Which statement best describes your health TODAY?
(ANXIETY / DEPRESSION )**

I am not anxious or depressed

I am slightly anxious or depressed

I am moderately anxious or depressed

I am severely anxious or depressed

I am extremely anxious or depressed

**29) How good or bad is your health TODAY?**

- This scale is numbered from 0 to 100.
- 100 means the best health you can imagine. 0 means the worst health you can imagine.
- Mark an X on the scale to indicate how your health is TODAY.
- Now, please write the number you marked on the scale in the box below.

YOUR HEALTH TODAY =

## Sickness absence

The following questions are about sickness absence.

*Sickness absence = working fewer hours or days than normally because of illness, injury or other health related reason.
Normal maternity leave is not considered sickness absence.*

**30) How many working days have you, all together, lost during the past 12 months due to sickness absence?**
*Please count only the days on which you would normally work. Also, partial sickness absence and therapeutic work count as sickness absence.*

None *(please continue with question 41)*

1 to 5 days (one working week)

5 to 10 days (one to two working weeks)

10 to 20 days (two to four working weeks)

More than 20 days (more than four working weeks)

**31) How many working days have you, all together, lost due to sickness absence during the last 6 months?**

None *(please continue with question 41)*

….. working days

**32) Have you, during the last 6 months, had a continuous episode of sickness absence of 5 days or more? (more than a week).**

No, I have not been absent during a continuous episode of 5 or more days (please continue with question 41)

Yes, 1 period *(please continue with question 33, 34)*

Yes, more than 1 episode, namely ….. episodes *(please continue with question 35)*

**33) What happened with your work during (or after) your episode of sickness absence?**

I have done the work myself, later, during normal working hours

I have done the work myself, later during extra hours.

My colleagues (or supervisor) took over my work during normal hours

My colleagues (or supervisor) took over my work during extra hours

Substitutes took over my work

My work has not been taken over

I do not know how my work has been taken over

**34) Can you make an estimation of how many extra hours it took to complete your work?**

….. hours *(please continue with question 43)*

**35) How many working days did you lose during the shortest episode of sickness absence of the last 6 months?**

….. days

**36) What happened with your work during (or after) your shortest episode of sickness absence?**

I have done the work myself, later, during normal working hours

I have done the work myself, later during extra hours.

My colleagues (or supervisor) took over my work during normal hours

My colleagues (or supervisor) took over my work during extra hours

Substitutes took over my work

My work has not been taken over

I do not know how my work has been taken over

**37) Can you make an estimation of how many extra hours it took to complete your work?**

….. hours

**38) How many working days did you lose during the longest episode of sickness absence durings the last 6 months?**

….. days

**39) What happened with your work during (or after) your longest episode of sickness absence?**

I have done the work myself, later, during normal working hours

I have done the work myself, later during extra hours.

My colleagues (or supervisor) took over my work during normal hours

My colleagues (or supervisor) took over my work during extra hours

Substitutes took over my work

My work has not been taken over

I do not know how my work has been taken over

**40) Can you make an estimation of how many extra hours it took to complete your work?**

….. hours

## Health care professionals and informal help

The following questions are about your use of health care and informal care during the past 6 months. With the help of these questions we want to inventory the health care professionals whom you have been seeing during the last 6 months. So please keep in mind the period between now and 6 months ago. It may be helpful to consult your diary, appointment cards, calendar etc.

*With the number of consultations, we mean surgery visits, telephone consultations, home visits etc. It can be helpful to consult your diary. If you do not know the exact numbers, you may make an estimation.*

**41) Did you consult your GP during the last 6 months? If you did, how many times?**

No

Yes, namely ….. consultation(s)

*(Please add all visits to the surgery, scheduled consultations, home visits and telephone consultations during the last 6 months.)*

**42) Did you have contact with an occupational health service during the last 6 months?**
*(E.g. an occupational physician, occupational health nurse, absentee controller etc.)*

No

Yes, namely the following number of contacts: .…..

*(Please add all contacts with the occupational health service: visits, scheduled consultations, home visits and telephone consultations during the last 6 months.)*

**43) Did you consult a movement therapist during the last 6 months?**
*(E.g. ergotherapist, physiotherapist, osteopath, chiropractor, Mensendieck, Cesar therapist etc.)*

No

Yes, namely the following number of contacts …..

*(Please add up all consultation visits, visits by appointment, home visits and telephone consultations over the past 6 months.)*

**44) Did you consult a medical specialist in the past 6 months?**
*(E.g. cardiologist, rheumatologist, internist, neurologist, etc.)*

No

Yes, namely the following number of contacts: …..

*(Please add up all consultation visits, visits by appointment, home visits and telephone consultations in the last 6 months.)*

**45) Have you had contact with a psychiatrist, psychologist or psychotherapist during the past 6 months without being admitted to an institution?**

No

Yes, namely the following number of contacts: …..

*(Please add up all consultation visits, visits by appointment, home visits and telephone consultations in the last 6 months.)*

**46) Did you have contact with alternative healers in the last 6 months?**
*(E.g.: acupuncturist, homeopath, physician, etc.)*

No

Yes, namely the following number of contacts: …..

*(Please add up all consultation visits, visits by appointment, home visits and telephone consultations in the last 6 months.)*

**47) Have you in the past 6 months been in contact with paid help from the professional home care? If so, do you want to indicate how many hours per week you had contact with professional home care?**

No

Yes, namely the following number of hours per week: …..

**48) In the past 6 months, have you received help from family or friends because of your state of health? If so, do you want to indicate how many hours per week you received help from your family or friends?**

No

Yes, namely the following number of hours per week: …..

**49) Have you been admitted to an academic or general hospital in the last 6 months? If yes, how many nights?**

No

Yes, namely the following number of overnight stays: …..

**50) Have you been admitted to a rehabilitation clinic in the past 6 months, other than in a hospital? If yes, how many nights?**

No

Yes, namely the following number of overnight stays: …..

**51) Have you been admitted for psychological treatment in the last 6 months, other than in a hospital? If yes, how many nights?**

No

Yes, namely the following number of overnight stays: …..

**52) Have you been in day treatment in the last 6 months? If yes, how many parts of the day? (By a half day we mean a morning or an afternoon; a whole day is two half days)**

No

Yes, namely the following number of day parts: …..

## Medicines

These are the last questions. These questions are about medicines that you have been prescribed by a healthcare provider, such as a general practitioner or psychiatrist, and medicines that are freely available, for example at the pharmacy or the drugstore or via the internet.

These questions are very important and we ask you to put your medication together so that the name and dose can be written down accurately.

**53) Have you used any medicines that have been prescribed by your doctor within the last 6 months?**
*(E.g.: clomipramine, 20mg; eletriptan, 40mg; Adalimumab (Humira), 50ml)*

No

Yes, namely ……………………………………………………………

*(Do not include medication during hospitalization, nor the contraceptive pill.)*

**54) Have you used any medicines that have not been prescribed by your doctor within the last 6 months?**
*(E.g.: alternative medicines such as herbs or nutritional supplements)*

No

Yes, namely ……………………………………………………………

**55) Have you used medical devices and adjustments in the home in the last 6 months?**
*(E.g.: wheelchairs, insoles, elevators, adapted worktops, etc.)*

No

Yes, namely ……………………………………………………………

**End of this questionnaire.
Thank you very much for your cooperation!**
