# Supplementary material for: Affecting patients with work-related problems by educational training of their GPs: a cost-effectiveness study
Source: BMC Fam Pract. 2019 Mar 2;20:38. doi: 10.1186/s12875-019-0924-9 (PMC6397438; doi:10.1186/s12875-019-0924-9)
Supplement: Supplementary file 2 — Supplementary Data Table S1. Description: Cost calculations per cost category (year of financial data = 2012). (DOCX 24 kb) [file 12875_2019_924_MOESM2_ESM.docx]

**Supplementary Data Table S1:** Cost calculations per cost category (year of financial data=2012)

**(i) Intervention costs**

The intervention costs were calculated using a bottom-up approach (see **table a.1**). In order to calculate the per patient intervention costs, the average number of working patients per GP practice (672,8) was multiplied with the number of GP practices included in the intervention (12) (per trainer approximately 6 GPs in one group, per training 12 GPs). It is important to note that when the number of working patients per GPs, or the number of GPs being trained, would increase, the total intervention costs would further decrease.

**Table a.1** Intervention costs

| **Type of cost factor** | **Calculation** | **Total Cost (€)** |
| --- | --- | --- |
| Personnel costs |  |  |
| Group training expert speaker  (N=2) | Time investment (10 hours)*hourly wage (€100) | 1,000 |
| Booster training expert speaker  (N=2) | Time investment (6 hours)*hourly wage (€100) | 600 |
| Preparation and travel time  (for 2 trainers) | Time investment (16 hours)*hourly wage (€100) | 1600 |
| ***Total personnel costs*** |  | ***3,200*** |
| Material costs |  |  |
| Binders and materials | Binders & materials (€15)*number of attendees and trainers (14) | 210 |
| Training location | (costs location group training €1,317.75)+(costs booster training €122.15/2) | 479.97 |
| ***Total material costs*** |  | ***689.97*** |
| ***Total intervention costs*** |  | ***3,889.97*** |
| **Per Patient Intervention Costs** | total intervention costs / patients being served by the intervention (N=8,074) | **0.48** |

**(ii) Healthcare costs**

Health care costs, covering care provider resource utilization (i.e. visits for primary and secondary care), home care, alternative care and medication, were calculated according to the guidelines for health care in the Netherlands by using the Dutch manual for costing ([1](#_ENREF_24)). Tariffs or an average price from providers were used when standard cost prices were not available. The costs of prescription medicines were calculated based on the price per dosage for medication costs in the Netherlands ([2](#_ENREF_25), [3](#_ENREF_26)). Over the counter medication prices were based on market prices (including 6% VAT) and medical and personal aids (e.g. orthotics, mobility aids, etc.) were based on costs per user within the aid category provided by the Dutch care institute ([4](#_ENREF_27)).

**(iii) Patient- and Family Costs**

Patient and family costs were incorporated by assessing informal care costs and travel and parking costs incurred while utilizing health care. Informal care was valued against the ‘shadow price’ (i.e. the price used when an official price is not available) of the wage rate per hour of a housekeeper. Travel costs were calculated by multiplying average distance with the standard price weights as derived from the Dutch manual for costing research ([1](#_ENREF_24)).

***(iv) Productivity costs***

Patient’s productivity was measured using the productivity and disease questionnaire (PRODISQ) ([5](#_ENREF_28)). The PRODISQ measures productivity loss from paid work (i.e. both absenteeism and presenteeism) in terms of total sick leave. Absenteeism refers to the total number of days lost from work and presenteeism refers to reduced work quantity and quality while at work ([5](#_ENREF_28)). Both absenteeism days and the inefficiency score were multiplied by the mean age- and gender wage rates of the Dutch population ([1](#_ENREF_24)). Productivity cost calculations were based on the friction cost approach which states that sick employees are replaced after a certain period of time called the friction period. After this friction period, no more lost productivity costs are assumed (6). The friction period of 92.68 days for the Netherlands was estimated based on the level of unemployment in 2012 (7).

**References Supplementary Data**

1. Hakkaart L, Tan SS, Bouwmans CAM. Manual for cost research. Methods and standard costs for economic evaluations in healthcare [In Dutch: Handleiding voor kostenonderzoek. Methoden en standaard kostprijzen voor economische evaluaties in de gezondheidszorg]. Institute for Medical Technology Assessment, Erasmus University Rotterdam, 2010. Available from http://www.zorginstituutnederland.nl
2. Healthcare institute Netherlands. Guideliness for pharamacoeconomic research, 2012. Available from: <http://www.zorginstituutnederland.nl>
3. Healthcare institute Netherlands. Drug costs in the Netherlands, 2014. Available from: <http://www.medicijnkosten.nl>
4. Healthcare institute Netherlands. GIP Databank for medical aids and medication, 2014. Available from: www.gipdatabank.nl
5. Koopmanschap MA. PRODISQ: a modular questionnaire on productivity and disease for economic evaluation studies. *Expert review of pharmacoeconomics & outcomes research.* 2005;5(1):23-8. doi:0.1586/14737167.5.1.23
6. Koopmanschap MA, van Ineveld BM. Towards a new approach for estimating indirect costs of disease. *Soc Sci Med.* 1992;34(9):1005-10.
7. Statistics Netherlands. Jobs; outstanding, new and filled. 2014. Available from: http://statline.cbs.nl/Statweb/
