# Supplementary material for: Affecting patients with work-related problems by educational training of their GPs: a cost-effectiveness study
Source: BMC Fam Pract. 2019 Mar 2;20:38. doi: 10.1186/s12875-019-0924-9 (PMC6397438; doi:10.1186/s12875-019-0924-9)
Supplement: Supplementary file 3 — “Ethics waiver” Description: Letter from the institutional ethics review board concluding that approval was not needed according to Dutch law. (DOCX 11 kb) [file 12875_2019_924_MOESM3_ESM.docx]

**Van:** Agt, Frans van
**Verzonden:** woensdag 6 april 2011 12:30
**Aan:** Kock, Kees de
**Onderwerp:** 2011/121 "Work in general practice"

Beste heer de Kock,

Ik ben u nog een antwoord schuldig op uw email van 18 maart 2011 over bovenstaand onderzoek. Namens de commissie kan ik u laten weten dat uw onderzoek niet onder de WMO valt. Voor de uitvoering is dan ook geen positief oordeel van de CMO vereist.

Met vriendelijke groet,

Mr dr Frans van Agt

CMO

**Translation:**

*Dear mr. De Kock,*

*I still owe you an answer in reaction to your email of march 18th 2011 regarding the research mentioned above. On behalf of the committee (CMO, Committee on Research inv. Human subjects KdK) I can let you know that your research is not covered by the Medical Research involving Human Subjects Act and/or the Embryos Act. Therefore, to proceed, no approval of the CMO is needed.*

*Kind regards,*

*Mr. Dr. Frans van Agt*

*CMO*
